# A novel distribution of supergene genotypes is present in the socially polymorphic ant *Formica neoclara*

**DOI:** 10.1186/s12862-022-02001-0

**Published:** 2022-04-13

**Authors:** Darin McGuire, Madison Sankovitz, Jessica Purcell

**Affiliations:** Department of Entomology, University of California, Riverside 900 University Ave, Riverside, CA 92507 USA

**Keywords:** Coadapted gene complex, Queen number, Formicinae, Population genetics

## Abstract

**Background:**

Supergenes are chromosomal regions with tightly linked clusters of alleles that control compound phenotypic traits. Supergenes have been demonstrated to contribute to the maintenance of polymorphisms within populations in traits as diverse as mimetic wing coloration in butterflies, mating strategies in birds, and malarial susceptibility in mosquitoes. A large supergene also underlies variation in social organization in *Formica* ants. Alternative supergene haplotypes are associated with the presence of either a single queen (monogyny) or multiple queens (polygyny) within colonies. Here, we assess the social structure and supergene status of the North American species *Formica neoclara*.

**Results:**

We sequenced a subset of the genome in 280 individuals sampled in populations from California to northern British Columbia using ddRADseq. We determined that *F. neoclara* is socially polymorphic in queen number, and we show that the social polymorphism is associated with alternative haplotypes at the social supergene. Intriguingly, polygyne colonies can harbor workers that are homozygous for both haplotypes as well as heterozygotes.

**Conclusions:**

This colony genetic composition contrasts with other *Formica* species, in which almost all individuals in polygyne colonies have the polygyne-associated haplotype. The social polymorphism is present in widely distributed and genetically subdivided populations of *F. neoclara.* In studying this system in *F. neoclara*, we expand our understanding of the functional evolution of supergene haplotypes as they diverge in different lineages.

**Supplementary information:**

The online version contains supplementary material available at 10.1186/s12862-022-02001-0.

## Background

Stable multilocus genetic polymorphisms often underlie complex phenotypic variation within populations [1–4]. Such coadapted gene complexes are present in many organisms [1], playing a role in mimicry in butterflies [1, 2, 5], mating morphs in birds [6, 7], and malaria susceptibility in mosquitoes [8]. These linked functional mutations, designated as supergenes, occur in regions of suppressed recombination [3, 9] that can act as a single Mendelian element when heterozygous [4, 5]. Supergenes allow for the unified control of compound phenotypes [3], providing a genetic mechanism to maintain balanced polymorphisms within populations [4]. A benefit of supergenes lies in their architecture; these clusters of tightly linked functional mutations often prevent disadvantageous intermediate phenotypes [4] through reduced recombination [3]. As supergenes are widespread, many organisms can serve as models of study. Supergenes have been explored in studies involving the evolution of phenotypic diversity, such as the divergence of geographic races of *Heliconius* butterflies [10]. Supergenes have also garnered scientific attention for their role in polymorphisms within populations [4, 11], including social organization in ant species [12–16].

Independent and distinct supergenes that underlie a polymorphism in colony queen number were initially described in two ant species, *Solenopsis invicta* and *Formica selysi* (Hymenoptera: Formicidae) [12, 14]. Monogyne colonies are headed by a single queen, whereas polygyne colonies have multiple queens, resulting in lower genetic relatedness among nestmates [17–19]. These large supergenes that span most of the chromosome were subsequently found in other *Formica* [15, 20] and *Solenopsis* species [16], meaning that they likely predate speciation of at least some species in these genera. Intriguingly, both supergene polymorphisms are partly maintained by selfish genetic mechanisms, but the precise mechanisms are different in each system. In *S. invicta*, the supergene haplotype found exclusively in polygyne colonies (Sb) selfishly promotes its propagation via a green-beard effect [13], in which heterozygous workers actively kill joining queens that lack the Sb haplotype [21]. Transmission ratio distortion at the supergene was also detected in *S. invicta* embryos, but this mechanism does not appear to consistently favor one haplotype over the other; instead, supergene-linked loci transmission ratios significantly differed from Mendelian ratios, with some queens producing more Sb eggs than expected and others producing more SB eggs [22]. The selfish genetic mechanism in *Formica selysi* also favors the polygyne-associated haplotype (Sp) through maternal effect killing [23], wherein offspring of heterozygous queens only survive if they have an Sp haplotype.

There are some notable differences between *Solenopsis* and *Formica* species in the distribution of supergene genotypes in colonies. In both cases, monogyne colonies contain exclusively one supergene haplotype, SB in *S. invicta* and Sm in *F. selysi* [12, 14]. In contrast, polygyne *S. invicta* colonies possess SB/SB and SB/Sb workers and SB/Sb queens. Sb/Sb females rarely reach adulthood [21, 24], potentially due to one or more deleterious alleles on the Sb social supergene [12]. Polygyne *F. selysi* colonies do not contain Sm/Sm homozygotes (workers or queens), but they do have Sp/Sp and Sm/Sp workers and queens [14, 19]. We are now beginning to look at the distribution of supergene haplotypes in other *Formica* species [15, 20]. Understanding the evolutionary history and any changes in the mode of action in supergenes found in multiple species will provide novel insights into the processes that shape complex phenotypic and multi-locus genetic polymorphisms.

*Formica neoclara* is an ant species found throughout western North America. Workers forage in trees [25], where they search for prey and tend honeydew-producing insects [26]. Past research has focused on the natural history [27] and agricultural relevance of *F. neoclara* [25, 26]. Despite its broad range and agricultural implications, the social organization and population structure of *F. neoclara* remain largely unknown.

Here, we investigate *F. neoclara* populations distributed from California to Northern British Columbia to determine whether the species is socially polymorphic and, if so, whether colony queen number is under genetic control throughout its range. Further, we investigate the genetic structure of populations across the range of this species to determine whether the population likely expanded recently or whether geographically distant populations are also genetically distant. Overall, this study will begin to uncover similarities and differences in social polymorphisms and their genetic bases in ant species with distinct evolutionary histories.

## Results

### Assigning social form to colonies

**Fig. 1:**
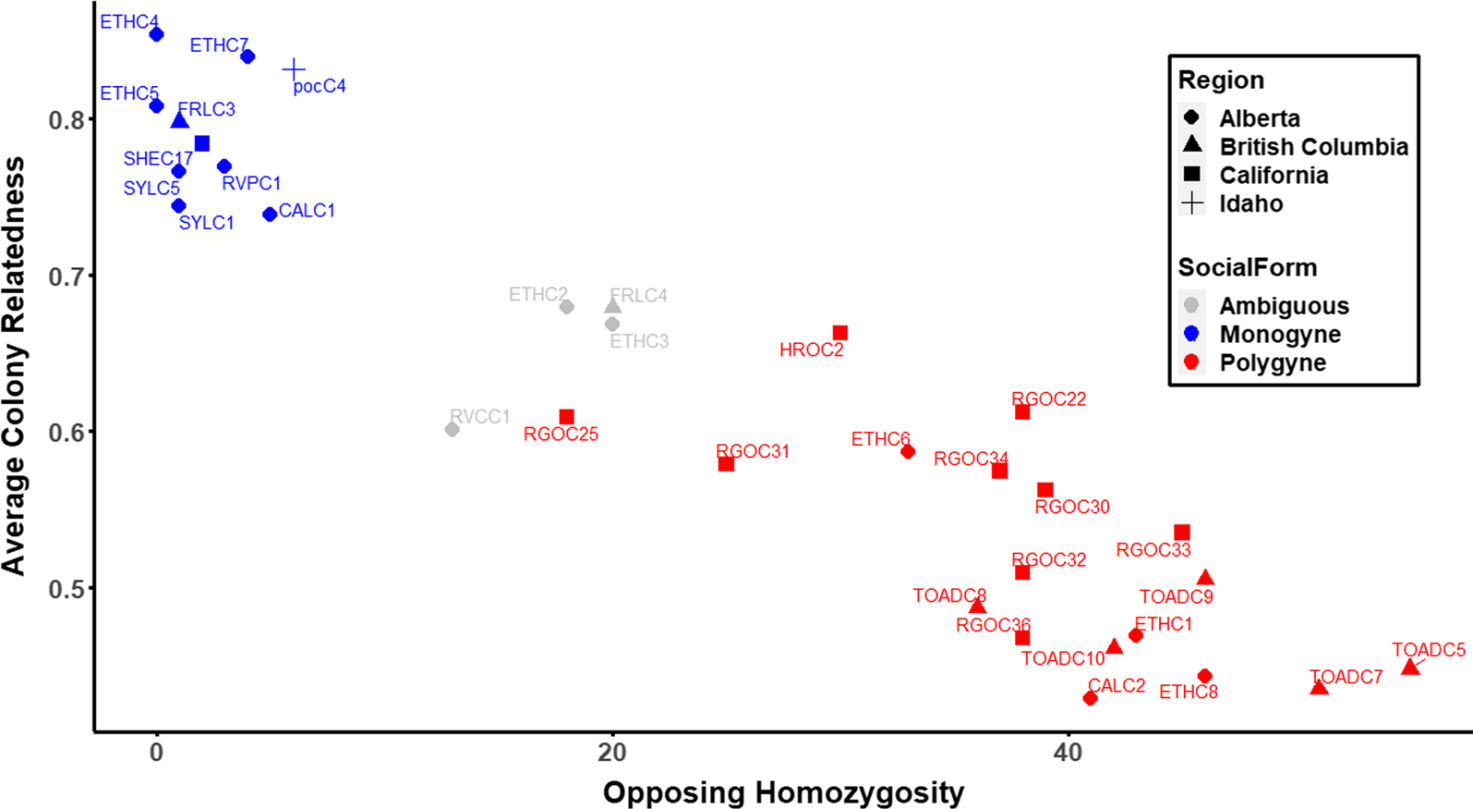
Scatterplot displaying colony-level metrics of opposing homozygosity and average relatedness among nestmates (Huang estimator). We infer that the upper left cluster contains monogyne colonies (blue), while the lower right cluster contains polygyne colonies (red). A few colonies are likely either monogyne and polyandrous or oligogyne (i.e. contained a small number of closely related queens), and we could not confidently assign their social structure. The “undetermined” (ambiguous) colonies are color-coded grey. These colonies are excluded from the GWAS analyses

Using multiple complementary metrics based on worker genotypes (based only on loci outside of chromosome 3), we determined the colony queen number and estimated mate number for 28 out of 32 colonies (Additional file 1: Table S1). Colonies with multiple queens generally had relatively high levels of opposing homozygosity and relatively low within-colony average relatedness estimates, while colonies with one singly-mated queen had little or no opposing homozygosity and high within-colony average relatedness (Fig. 1). In several cases, intermediate opposing homozygosity and average relatedness values, along with parentage inferences from COLONY, suggested that colonies either contained one multiply-mated queen or several related queens. When metrics conflicted with one another, we labeled these colonies as ‘undetermined.’ To be conservative in downstream analyses, we excluded these colonies from the GWAS analysis.

### Identifying supergene genotypes of individuals

**Fig. 2:**
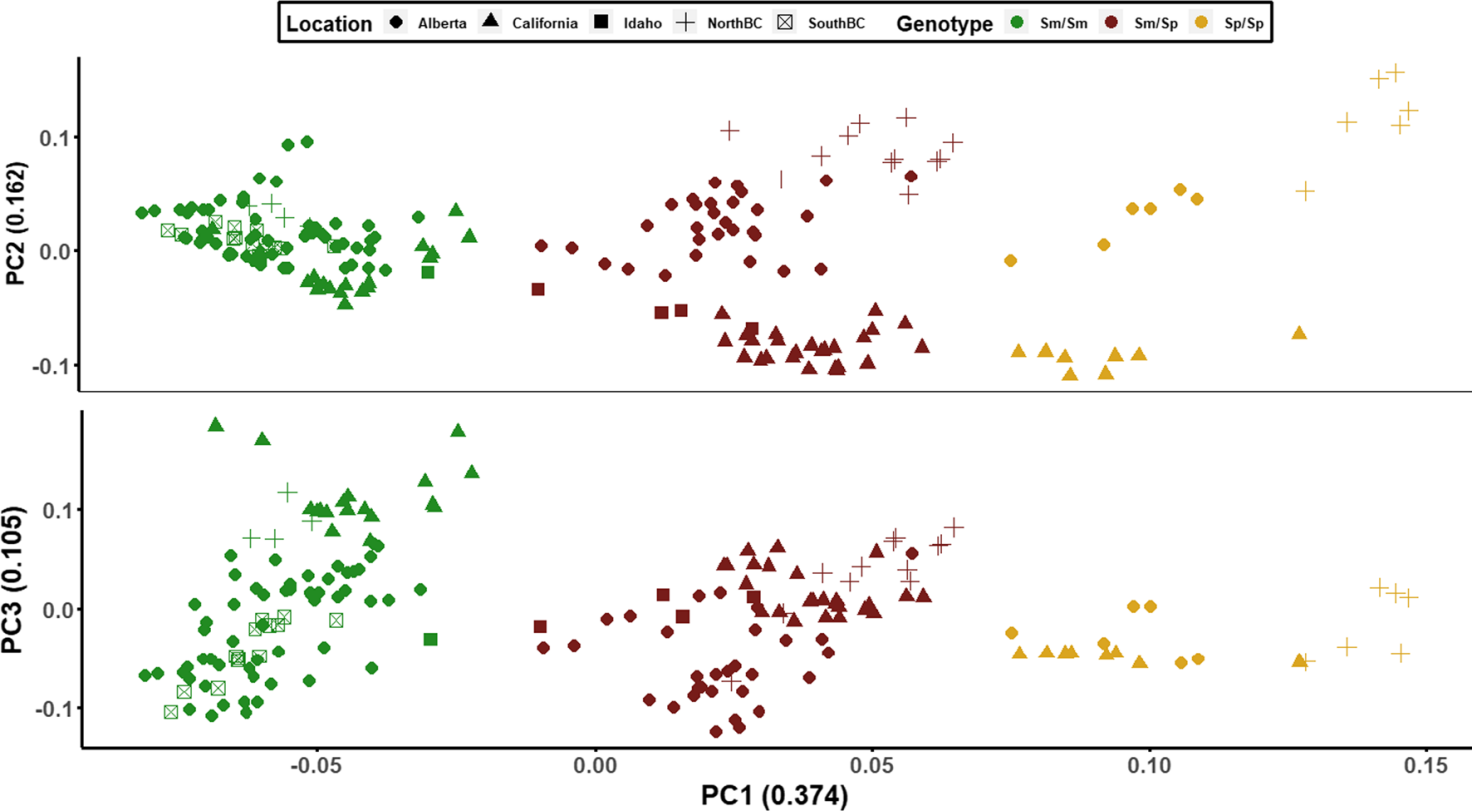
Principal component analysis (PCA) for the low-recombining region of chromosome 3. The PCAs compare principal components 1 (PC1) and 2 (PC2) (**A**), and 1 (PC1) and 3 (PC3) (**B**). Principal component 1 distinguishes three genotypes on chromosome 3. Negative *F*_*IS*_ values (based on markers on chromosome 3) distinguish individuals that are heterozygous for the supergene (brown cluster) from the homozygotes (green and yellow clusters, positive F_IS_ values). To verify which cluster is homologous with the Sm/Sm genotype in *F. selysi*, we compared the three genotypes to the *F. selysi* reference allele (Sm). Individuals in the green cluster tend to be homozygous for the reference allele across the supergene, suggesting that they are Sm/Sm. Thus, the yellow clusters of homozygotes have region-specific versions of the Sp/Sp genotype. Principal component 2 shows geographic structure in the Sp haplotype, while PC3 shows geographic structure in the Sm haplotype

Independent of the social structure assessment, we determined whether there were long-range haplotypes in *Formica neoclara* in the 2–12.5 Mbp region of chromosome 3, which contains the social supergene in *F. selysi* [14]. We performed a principal component analysis (PCA) for all individuals using 26 markers from the *F. selysi* supergene region. Along PC1, in particular, we observe three distinct clusters of individuals. Individuals shown in brown are in the central cluster along PC1 and have excess heterozygosity, as determined by negative F_IS_ values on chromosome 3, suggesting that they are heterozygous for two distinct supergene variants. Of the two remaining clusters, the alleles of individuals in the leftmost cluster align consistently to the *F. selysi* reference alleles, which is based on monogyne males with the *F. selysi* ‘Sm’ supergene haplotype (green cluster, Fig. 2). The rightmost cluster more often is homozygous for the alternate allele (yellow cluster, Fig. 2). On this basis, we inferred that individuals in the left cluster (green) are homozygous for the *F. neoclara* Sm, individuals in the center cluster (brown) are heterozygous (Sm/Sp), and individuals in the right cluster (yellow) are homozygous for the alternative haplotype (Sp). Looking across PC2 and PC3, we observe signatures of geographic variation within each supergene genotype cluster (Fig. 2, shapes).

**Fig. 3:**
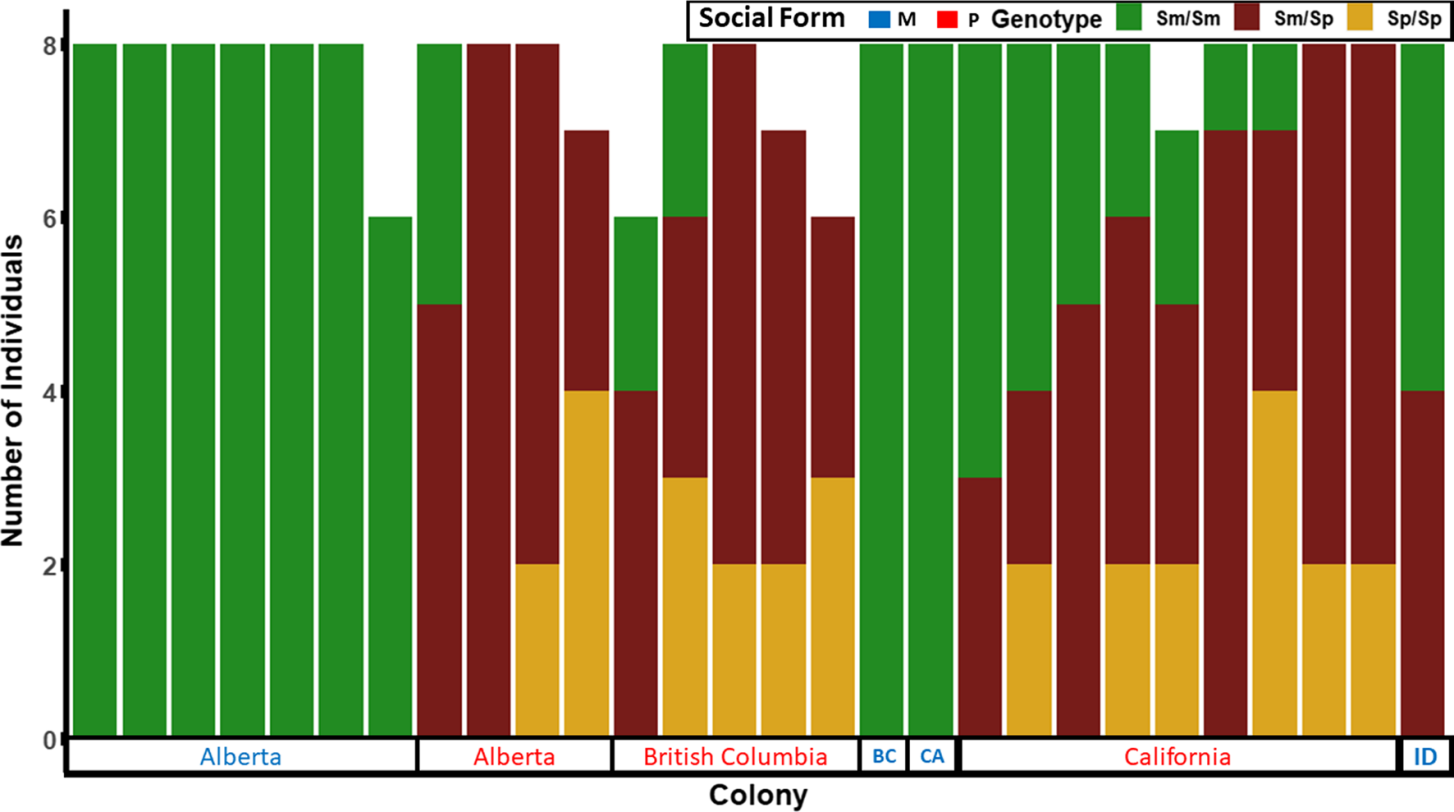
Stacked bar plot displaying genotypes of samples from colonies ordered by region and haplotype frequency. Each bar represents all samples from an individual colony. Sampling regions include Alberta, northern British Columbia (British Columbia), southern British Columbia (BC), California (CA), and Idaho (ID). A blue region name indicates that the colonies are monogyne, while a red region name indicates that colonies are polygyne. Genotype is indicated by color: green = Sm/Sm, brown = Sm/Sp, and Yellow = Sp/Sp. Genotype (green, brown, yellow) is shown for each individual worker, while phenotype (red, blue) relates to the colony as a whole. Colonies with “undetermined” social structure were excluded from this analysis (N = 4; see Additional file 1: Fig. S3)

In complement, we performed a genome-wide efficient mixed model association (GEMMA) to identify single nucleotide polymorphisms (SNPs) associated with variation in colony queen number. When we restrict the analysis to the socially polymorphic Alberta population (85 individuals from 11 colonies), we see a strong association between five SNPs on chromosome 3 and colony social form (the presence or absence of multiple queens, inferred by *COLONY*). These SNPs lie between 7.7 Mb and 12.6 Mb (Additional file 1: Fig. S1), and they are a subset of the 11 markers that contributed most strongly to observed variation along PC1 (Fig. 2, Additional file 1: Table S3). We detected no significant SNPs elsewhere in the genome. When we analyze the data of all populations with an inferred social structure (215 individuals from 28 colonies), we detect one significant SNP on chromosome 3 at 12.1 Mbp. We posit that the genetic variation between populations affects the signal-to-noise ratio of the latter analysis. In addition, the presence of Sm/Sm homozygous workers in polygynous colonies (Fig. 3) and the low marker density in our dataset influence the power of these statistical analyses.

### Supergene genotypic distribution within regions and colonies

Both monogyne and polygyne colonies were found across the broad geographical sample tested in this study. Every colony independently determined to be polygyne harbors at least three workers with the Sp haplotype. Interestingly, all polygyne colonies have Sm/Sp individuals present and frequently contain individuals with all three supergene genotypes (Sm/Sm, Sm/Sp, Sp/Sp). In contrast, all but one colony determined to be monogyne are composed of Sm/Sm workers exclusively. In one exception, the solitary colony from Idaho (PocC4) contains both Sm/Sm and Sm/Sp workers, and we deemed this colony to have one multiply-mated queen on the basis of the low opposing homozygosity and high relatedness values (although COLONY results suggested that two queens may be present). This phenotype call could be an error; we expect relatedness estimates to be inflated when local samples sizes are small. In parallel, workers from PocC4 exhibited an elevated F_IS_ value relative to the population average, suggesting that the mother queen would have had particularly few heterozygous loci. For single polyandrous queens, the opposing homozygosity value will be limited by the number of heterozygous loci in the queen.

**Fig. 4:**
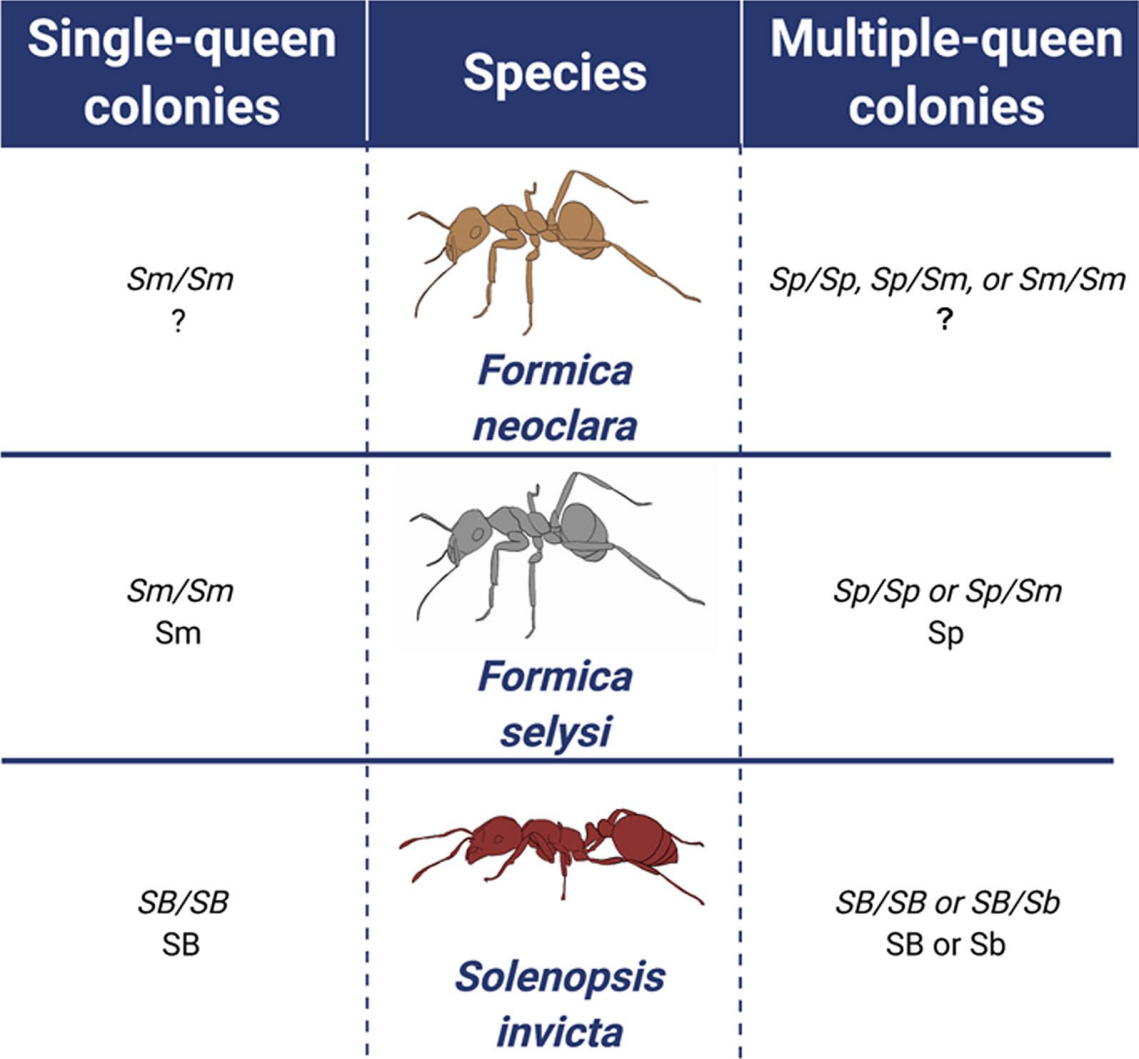
The genetic system underlying social organization in three ant species with the social supergene [12, 14]. Offspring genotype possibilities are shown; italicized genotypes are female and non-italicized genotypes are male

Overall, colonies from Alberta, British Columbia, and California possess the three respective genotypes: Sm/Sm, Sm/Sp, and Sp/Sp. Although the Sm/Sm genotype is most common in monogyne colonies, it is also found in polygyne colonies (Fig. 3). The presence of all three genotypes in polygynous *F. neoclara* colonies contrasts with previously studied ant species with social supergenes (Fig. 4).

### Population genetic structure

**Fig. 5:**
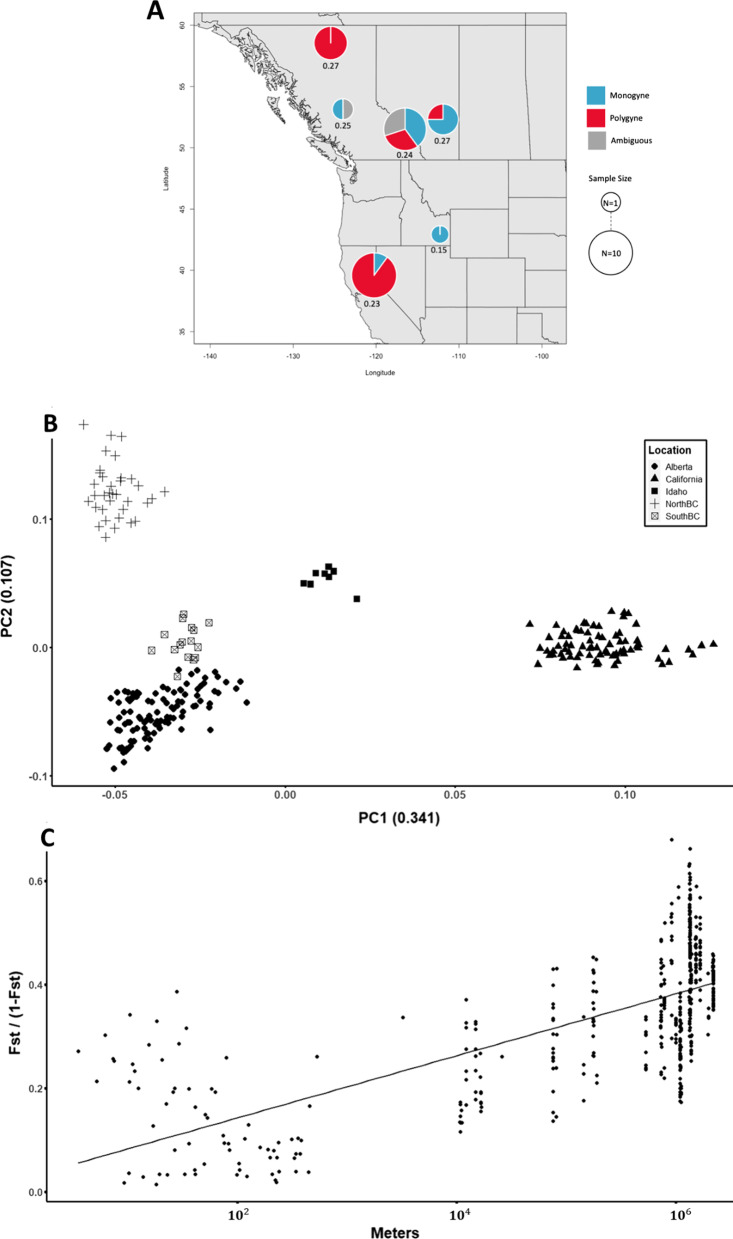
**A** Location, social form, and expected heterozygosity of the six regions spanning the sample sites. Pie charts show the proportion of monogyne, polygyne, and undetermined colonies in the population, with the pie chart size indicating sample size. Expected heterozygosity is shown below each pie chart. **B** A PCA without chromosome 3 data showing principal components 1 (PC1) and 2 (PC2) and their respective weights in parentheses. Clusters appear to be based on geographic location. Point shapes are determined by sample location; circle = Alberta, triangle = California, filled square = Idaho, cross = Northern British Columbia, open square = Southern British Columbia. **C** A scatter plot displaying isolation by distance (IBD). Each point represents a pairwise comparison between two colonies. The linear geographic distance between the two colonies (in meters) is on the x-axis, and Rousset’s [53] distance is on the y axis

Expected heterozygosity values from Alberta (sites from the plains and Rocky mountains grouped separately in the map), northern and southern British Columbia, California, and Idaho range from 0.15 to 0.27, with an average of 0.235 (Fig. 5A). This pattern is not consistent with a recent population expansion in these parts of the species range. The PCA utilizing all markers except those on chromosome 3 reveals clustering by region of origin as well, further supporting the inference that these populations are genetically distinct (Fig. 5B). Isolation by distance (IBD) analysis utilized the 32 colonies with six or more worker samples. The pairwise F_ST_ values between colonies ranged from 0.014 to 0.405, with a mean of 0.245 (Fig. 5C). Pairwise distances between colonies were also variable, ranging from 3.54 m up to 2200 km, with an average of 964 km. The r^2^ for geographic distance by genetic distance is 0.408 (p < 0.0001).

## Discussion

*Formica neoclara* exhibits a social polymorphism in queen number across its range. The supergene underlying queen number variation in multiple *Formica* species [15] is also present and associated with colony queen number in *F. neoclara.* However, the distribution of haplotypes within nests is notably different from other previously studied *Formica* species (Figs. 3 and 4). In both *F. selysi* and *F. neoclara*, individuals in single queen colonies are all homozygous for the monogyne-associated haplotype, Sm. The difference between the species is observed in polygyne colonies. In *F. selysi*, every individual in a multiple queen colony harbors at least one copy of the polygyne-associated haplotype Sp (queen and worker genotypes include Sp/Sm and Sp/Sp) [14, 19]. In contrast, *F. neoclara* polygyne colonies can harbor individuals lacking the Sp allele, with some colonies containing all three possible genotypes (Sm/Sm, Sm/Sp, and Sp/Sp). Out of 18 polygyne colonies sampled, we never detected a multiple queen colony with exclusively Sm/Sm individuals. This pattern suggests that the association between the supergene and colony queen number is present in *F. neoclara*, as in other *Formica* species, despite differences in haplotype distribution within colonies.

The distribution of genotypes within polygyne colonies raises questions about how the genetic and phenotypic polymorphisms are maintained in *F. neoclara.* Finding Sm/Sm workers in polygyne nests in all populations suggests that the ‘maternal effect killing’ selfish genetic mechanism found in *F. selysi* is not operating in *F. neoclara* [23]. Based on the limited information available in other systems, we cannot yet determine whether the selfish genetic mechanism evolved recently in the *F. selysi* clade or whether it was lost from the *F. neoclara* clade. Preliminary evidence from a small number of colonies suggests that *F. cinerea*, a close relative of *F. selysi*, may have Sm/Sm genotypes in polygynous colonies in some populations [15]. A similarly small sample of polygynous colonies of *F. glacialis*, a relative of *F. neoclara*, detected no Sm/Sm workers [28]. Studies of additional species across the genus are needed to trace the evolutionary history of the maternal effect killing mechanism. Likewise, finding Sp/Sp workers in polygyne nests suggests that the Sp haplotype may not contain highly deleterious alleles. In the convergently-evolved fire ant supergene, the polygyne-associated haplotype, Sb, has highly deleterious alleles, such that Sb/Sb individuals almost never survive to adulthood and reproduce [12, 21, 29, 30]. Further research is needed to understand what selective pressures maintain the genetic polymorphism and prevent either haplotype from sweeping to fixation in these populations. One possibility may be that the genetic and phenotypic polymorphisms evolved or are maintained through spatially heterogeneous selection, in which each social form experiences advantages in different environments. This spatially variable selection would prevent fixation of either form, and previous studies have identified differences in the distribution of monogyne and polygyne *F. selysi* colonies at both local [31] and regional scales [32]. However, there is no clear gradient in the distribution of social forms along the large latitudinal gradient covered by our sampling effort (Fig. 5A). Given that some of the well-studied mechanisms found in *S. invicta* and *F. selysi* appear to be weak or absent, further research is needed to determine what factors maintain this genetic polymorphism in *F. neoclara*.

GEMMA analysis of all populations reveals that at least one SNP on chromosome 3 (12.1 Mbp) is associated with social form. Restricting the analysis to colonies from Alberta shows that five SNPs on chromosome 3 are correlated with social form. A handful of genes associated with or around regions of the supergene (chromosome 3) are conserved across multiple *Formica* species, with *Knockout* standing out as a strong candidate gene [15, 20]. None of the markers used in this study were positioned in *Knockout* or in other candidate genes identified by previous studies [20], but the marker density was low in the present study.

To assess colony social structure, we employed a method that evaluates the opposing homozygosity of biallelic RAD loci and the nestmate relatedness in parallel (Fig. 1). Members of our team have used variations of this method in several other species (Pierce et al., in revision) [28], but the present study spans the most massive spatial scale. Most sampled colonies either exhibited a relatively low number of opposing homozygotes and a high level of relatedness, suggesting that workers are all daughters of a single queen, or had high opposing homozygosity paired with low relatedness, suggesting that workers are produced by multiple queens. Overall, we propose that combining these methods complements more standard assessments of colony-level relatedness alone and parentage inference tools, implemented in programs like COLONY (and we used these methods as well, Additional file 1: Table S1). We lay out the benefits and drawbacks of opposing homozygosity and relatedness in this dataset, and we suggest that the combination of the two reduces biases associated with missing data and population structure.

We first note that both opposing homozygosity and relatedness result in some ambiguity at intermediate values. Specifically, intermediate levels of opposing homozygosity and average relatedness can be found in two types of colonies: those containing a polyandrous single queen or a small number of related nestmate queens (i.e. oligogynous colonies). We assessed the distribution of relatedness values in colonies determined to be monogyne, undetermined, and polygyne from the same population (Additional file 1: Fig. S4). The pairwise relatedness values for undetermined colonies exhibit a bimodal distribution that could indicate a mix of full and half siblings (i.e. offspring of a polyandrous single queen) or a mix of full siblings and cousins (i.e. offspring of two sister queens). We highlight two potential sources of error or bias in opposing homozygosity based on RADseq data. In theory, we should never detect opposing homozygosity in workers produced by singly-mated monogyne queens. However, we note that rare genotyping errors (especially non-detection of one allele in truly heterozygous individuals) can generate a small number of loci that exhibit apparent opposing homozygosity. Second, while opposing homozygosity should be robust to population structure, the maximum opposing homozygosity value for a single polyandrous queen will be based on the number of heterozygous loci in that individual. If queens vary in their level of inbreeding, this could result in variation in the maximum observed opposing homozygosity among populations. This issue would be most severe in colonies with a single polyandrous queen. Opposing homozygosity counts in polygynous colonies are determined by the genotypes of multiple queens, so are not dependent on the observed heterozygosity of the queens. A drawback of measuring relatedness in highly subdivided populations is an upward bias in relatedness estimates within relatively remote or undersampled populations. Both methods are sensitive to missing data, but we removed individuals with high levels of missing data from these analyses to account for this bias.

Overall, polyandry likely occurs at a relatively low frequency in *F. neoclara*, as has been detected in *F. selysi* [19] as well as *F. aquilonia* [33] and *F. truncorum* [34]. We expect that the four colonies classified as “undetermined” are most likely monogyne colonies with a polyandrous queen. This is consistent with the COLONY parentage inference for these colonies (Additional file 1: Table S1). However, as stated in the previous paragraph, both opposing homozygosity and relatedness values would be similar in oligogynous colonies, and COLONY inferences are not always reliable [35]. With three exceptions, colonies called as monogyne or as polygyne were supported by COLONY inference, relatedness metrics, and opposing homozygosity. In all exceptions (PocC4, ETHC6, and CALC2), the COLONY inference deviated from other metrics, but other metrics aligned with our social structure designation.

There are several other facets of our dataset that could influence the classification of parentage in colonies from our dataset. First, our sample covered a large geographic scale, but *F. neoclara* population densities tended to be low. As a result, some geographically isolated sites were represented with just a single colony in our dataset. As stated previously, relatedness values for colonies within populations with few samples were biased upward. For example, two relatively isolated colonies, PocC4 and HROC2, appear to display elevated relatedness values. On a technical note, our dataset also includes individuals sequenced in single-end and paired-end reads in different batches (Table 1). However, we used conservative filters to retain loci that were sequenced in all three batches and verified that there was no pervasive batch effect in the data used in our analyses (Additional file 1: Fig. S5).

The principal component analysis using chromosome 3 markers revealed some population structure in both the Sp and the Sm haplotypes at the continental scale. Performing a principal component analysis for all markers except those on chromosome 3 yielded strong signals of geographic population structure. We see distinct clustering by region, with principal components 1 and 2 apparently separating the clusters by latitude and longitude, respectively (Fig. 5B). This structure, combined with the discoveries that genome-wide expected heterozygosity is high across our spatially distant localities and F_ST_ is elevated between populations, suggests that these populations likely have a long history of independence, with gene flow occurring rarely or slowly at this scale. Given the latitudinal distribution of our sampling sites, from 39.3° N to 58.8° N, we initially expected that we might find evidence of a recent expansion from one or more southern refugia following the last glacial maximum. Instead, we see no clear latitudinal pattern in the distribution of expected heterozygosity and population differentiation, with expected heterozygosity values relatively homogeneous across populations (Fig. 5A). Additionally, most colonies display elevated pairwise F_ST_ values, save for pairwise comparisons of polygyne colonies in California and Northern British Columbia, which are in close proximity to neighboring colonies within their respective regions. Monogyne colonies, even when in close proximity, tend to display elevated F_ST_ values. Within our sampled colonies, at least some allele frequency variance between populations is explained by geographic distance (Fig. 5C). In *F. selysi*, patterns of isolation by distance suggest restricted dispersal for queens but not males [19]. However, within many *Formica* species, strong patterns of isolation by distance appear to be uncommon [36]. Future studies should investigate the genetic and phenotypic differences between the geographic variants of the Sm and Sp haplotypes using higher marker densities and additional field collection. This investigation would provide an ideal opportunity to understand how the evolutionary trajectories of supergene haplotypes, which differ in the effective population size and, potentially, mode of transmission, diverge within a widespread species.

## Conclusions

*Formica neoclara* is socially polymorphic in queen number across its broad geographic range. This polymorphism is associated with divergent haplotypes at the previously identified *Formica* social supergene. Interestingly, polygyne colonies frequently harbor Sm/Sm workers, a pattern that has not been previously identified in other species with the *Formica* supergene. As a result, this system offers a promising opportunity to examine epigenetic differences based on genotype and, independently, social origin, at least for Sm/Sm individuals. In conclusion, our study clearly shows a novel axis of variation in the evolution of the *Formica* supergene: haplotypes must have some functional differences among species, despite sharing a common evolutionary origin.

## Methods

### Field sampling, DNA extraction, and sequencing

We collected *F. neoclara* workers from colonies and along transects in Alberta, British Columbia, California, and Idaho in June–July, 2016. Whenever possible, we sampled at least eight workers from each colony. The transects consisted of collecting the first *Formica* ant that we observed every hundred meters along a road or trail in a chosen location, up to eight individuals. We frequently sampled individuals from different species at each stop along the transect. We stored samples in 100% ethanol.

We extracted DNA from the head and thorax of workers using a QIAGEN DNeasy Blood & Tissue Kit, following the insect tissue protocol with several modifications. Specifically, we manually ground the tissue in a tube while immersed in liquid nitrogen, used alternatively sourced spin columns (BPI-tech.com), 70% ethanol for the second DNA wash, and eluted the DNA in 30 µL of buffer AE. We then used a double-digest restriction site associated DNA sequencing (RADseq) approach to sequence samples (for protocol, see [37]). Briefly, we digested the DNA using restriction enzymes MseI and SbfI and ligated barcoded adapters. Next, we removed small DNA fragments using a mix of Sera-Mag SpeedBeadsTM Magnetic Carboxylate-Modified Particles (Thermo Fisher Scientific, cat. #65152105050250) and PEG/NaCl buffer [38]. We then amplified each sample in four separate PCR reactions, pooled all PCR products, and did a final round of small fragment removal using the Sera-Mag bead mixture. We sequenced 288 ant workers (8 were technical replicates of one colony, and we removed them from subsequent analyses) in three pooled libraries containing additional samples of other species not used in this analysis (Table 1).

**Table 1:** Overview of libraries and samples

Library year	# *F. neoclara* samples and # of total samples in the library	Sequencing facility	Sequence information	Average mean depth per individual, after filtering (range)
2016	75 of 1368	UC Berkeley Genomics Core	100 bp single-end reads, Illumina HiSeq 4000	123.0× (range 13–162.7)
2017	125 of 2629	UC Berkeley Genomics Core	150 bp paired-end reads, Illumina HiSeq 4000	68.1× (range 13.3–153.4)
2019	80 of 2348	Novogene	150 bp paired-end reads, Illumina HiSeq X 10	66.4× (range 13.8–134.1)

### Bioinformatics

We demultiplexed reads across each of the three batches using the process_radtags (version 1.4) command in Stacks, with default parameters [39]. To merge paired-end reads and remove the adapter sequence, we used *PEAR* [40]. We then aligned reads to the *Formica selysi* reference genome [15] using *BWA* and called genetic variants across the sample using *BCFtools mpileup* [41].

We initially filtered genotypes using *VCFtools* (v 0.1.13) [42] for missing data to remove genotype calls based on insufficient read depth (*--minDP* 7), to remove loci that were present in fewer than 80% of samples (*--max-missing* 0.8), and to remove sites with a minor allele frequency less than 0.05 (*--maf 0.05*). Samples with more than 20% missing data were removed prior to analysis. Batch effects were evident with more permissive max-missing thresholds, but the threshold of 80% ensured that retained loci were present in all three sequencing batches (Additional file 1: Fig. S5). The individual missingness threshold was determined to minimize the inflation of relatedness values of workers within colonies (Additional file 1: Fig. S2). This filtering resulted in 342 retained loci in 280 workers.

We assessed colony composition using multiple metrics, allowing us to come to a consensus to infer colony queen number. To ensure that these analyses were independent of our assessments of supergene variation, these analyses excluded all markers on chromosome three. The *COLONY* program [43] allowed us to infer the queen number of 32 colonies. We separated colonies by region (Alberta, California, British Columbia, Idaho) and ran *COLONY* once for each region. We excluded colonies with fewer than six workers (three in total: GCRC7, BHSC2, FRLC6) from colony-level analyses. After inferring queen number, we estimated the average relatedness among workers for the 32 colonies using several estimators. Relatedness calculations include the Ajk statistic (--relatedness) [44] and kinship-based inference for genome-wide association studies (KING) ɸ (--relatedness2) [45] available with *VCFtools* as well as the Huang diploid A estimator available on the PolyRelatedness program (e 14 0) [46]. The unadjusted Ajk statistic is the genomic relationship of each pair of subjects j and k, calculated from SNPs. Estimates of relationships use individuals in the sample as a base so that the average relationship between all pairs of individuals is 0. The expectation for output values is 0 for individuals within populations and 1 for individuals within themselves [44]. KING uses only markers with genotype data for both individuals, outputting kinship coefficients, ɸ. Values of ɸ have a maximum of 0.5, with values above 0.354 being considered duplicates or monozygotic twins [45]. The Huang estimator uses a method of moments approach, equating sample moments with population moments to output pairwise relatedness values. Several factors can decrease the certainty of relatedness estimator values [46]. Therefore, we used these three relatedness estimators jointly to account for shortcomings within the individual estimators. Rare variants can impact the Ajk statistic: allele frequencies near 0 or 1 make the equation unstable. ɸ loses reliability when individuals are from a mix of close and distant populations [45], which can be an issue in large geographic scale analyses such as this. We show that the Huang estimator is impacted by missingness, with individuals with higher levels of missing data inflating their own and population mean pairwise relatedness estimations (Additional file 1: Fig. S2). In addition to these relatedness estimators, we calculated the pairwise proportion of identity by descent between individuals (plink --genome, v1.07) [47]. Finally, we used opposing homozygosity to infer whether colonies have two or more parents (following [28]). We calculated opposing homozygosity for the respective colonies by counting the loci for which homozygotes were present for both the reference and alternative alleles within a colony, for bi-allelic single-nucleotide polymorphisms (SNPs). We inferred monogyne colonies as those with one queen identified by COLONY, higher average relatedness, and lower opposing homozygosity. We inferred polygyne colonies as those with more than one queen identified by COLONY, lower average relatedness, and higher opposing homozygosity. When multiple estimators resulted in conflicting signals, we considered the colonies to have an undetermined (‘ambiguous’) social structure.

We assessed the association between the social polymorphism and the supergene region using two complementary approaches. First, after determining the colonies’ putative social form, we assessed the supergene genotypes of individuals within said colonies. We assigned genotypes based on their position on a principal component analysis (PCA) of markers from chromosome 3 (Fig. 2). The known region of suppressed recombination on chromosome 3, which spans from 2 to 12.5 Mbp in *F. selysi* [20], was analyzed in *plink* (*--pca --allow-extra-chr*) [47] (n = 26 loci). We determined that individuals within a colony having an inbreeding coefficient, F_IS_, value above zero were homozygous, while those with a F_IS_ value below zero were heterozygous (--het, *VCFtools*). Individuals within the center cluster on the PCA (Fig. 2, brown points) are all heterozygous within the low recombining region, based on negative F_IS_ values. To distinguish the putative Sm/Sm and Sp/Sp homozygotes, we compared the SNPs of individuals from each cluster to the *F. selysi* Sm reference genome. One group of homozygotes had a higher proportion of reference alleles and was determined to represent the Sm/Sm workers. Based on clusters in the PCA of the low recombining region of chromosome 3 and an assessment of heterozygosity, we assigned genotypes to individual *F. neoclara* workers.

Second, we performed a genome-wide efficient mixed model association (GEMMA) analysis to test for an association between each locus and the inferred social form of each colony. This GWAS was performed independently of genotype assignment, using only social form assignment and markers on chromosome 3. We ran two analyses: one for colony samples from all regions (N = 215 individuals included) and one for colonies from Alberta, Canada only (N = 85 individuals). The latter analysis reduced the effects of population structure on the analysis. We excluded workers from transect samples and colonies labeled as ambiguous in social form from these analyses (41 individuals in total). *Beagle* (v 5.1) [48] was used to impute missing genotypes within the *F. neoclara* genetic data. *GEMMA* [49] was used to estimate a relatedness matrix (-gk 1) and then fit a linear mixed model to each SNP (*-k -lmm 1*). We then visualized output data from this process via a Manhattan plot (Additional file 1: Fig. S1).

To observe whether the geographically distant populations show signs of historic isolation or recent expansion, we utilized SNPs not on chromosome 3 for multiple analyses. We calculated expected heterozygosity at variable sites (--site-pi, *VCFtools*) for each population as the average nucleotide diversity per variable site on all chromosomes except chromosome 3 of one individual per colony (the individual with the least missing data, ranging from 0 to 9.32%) and all transect samples. Following this, we performed a PCA using all markers except those on chromosome 3 (N = 311, *plink --pca --allow-extra-chr*). Lastly, we performed a pairwise isolation by distance (IBD) analysis on the 32 colonies. Like previous colony-level analyses, we excluded colonies with fewer than six individuals. We calculated the Weir and Cockerham F_ST_ [50] between each colony using the --weir-fst-pop command in *VCFtools*. We calculated the distance between colonies using the *Imap* package (v1.32) [51] in R [52]. We then plotted the linear geographic distance by Rousset’s [53] genetic distance (Fig. 5C).

## Supplementary Information

**Additional file 1: FigureS1.** **A** Results of GEMMA analysis utilizing workers from colonies fromall regions, visualized via Manhattan plot. We used a linear mixed model withcolony social form as the independent variable. Each point represents anindividual SNP, with the corresponding chromosome on the x-axis and thenegative logarithm of the SNP p-value on the y-axis. Only one SNP, from chromosome 3, exceeds the significance level (Bonferroni corrected significance threshold: 1.52E−04;p-value: 7.26E−06). **B** Results of GEMMA analysis onworkers from colonies in Alberta only, to reduce the effect of underlyingpopulation structure on the GWAS, visualized via Manhattan plot. We used alinear mixed model with colony social form as the independent variable. Eachpoint represents an individual SNP, with the corresponding chromosome on thex-axis and the negative logarithm of the SNP p-value on the y-axis. Five SNPs,all from chromosome 3, are above the significance threshold (Bonferroni corrected significance threshold: 1.89E−04; p-values: 7.79E−07, three at 4.43E−07, and 3.79E−07). **Figure S2.** Stacked bar plot displaying genotypes of samples from colonies labeled as ambiguous in social form (three from Alberta, one from southern British Columbia). Each bar represents all samples from an individual colony. Genotype in relation to each individual worker, is indicated by color: green = Sm/Sm andbrown = Sm/Sp. **Figure S3.** Line graph showing the effects of missingness on the Huang estimator. Individual (red) and whole sample (black) relatedness values are represented as lines. **Figure S4.** Density plot showing pairwise relatedness (determined by PolyRelatedness) among all sequencedworkers of colonies from the Evan Thomas trailhead in Alberta, Canada. On average, workers from monogyne colonies are the most related, whereas intermediate colonies (“ambiguous” in Fig. 1) have a bimodal distribution offull siblings and individuals with intermediate relatedness values, andpolygyne colonies have the lowest overall pairwise relatedness. **Figure S5.** A principal component analysis utilizing all markers except those on chromosome 3 in individuals fromcolony samples, with the year of the respective batches color coded. **Table S1.** Overview of consensus social form variables. **Table S2.** Sampling regions and their associated coordinate values. **Table S3.** PC axis weightings of variants on chromosome 3 (see also Fig. 2). Weightings greater than 1 or less than − 1 are highlighted in grey for each PC axis. The outliers from GWAS analyses are highlighted in green (Alberta only) and orange (GWAS of the full dataset).

## Data Availability

The datasets generated and analyzed during the current study are available in the NCBI SRA, https://www.ncbi.nlm.nih.gov/bioproject/815367. Sample locality data is available in Additional file 1: Table S2.

## Abbreviations

PCA: Principal component analysis
PC: Principal component
GEMMA: Genome-wide efficient mixed model association
SNP: Single nucleotide polymorphism
IBD: Isolation by distance
Sm: Monogyne-associated haplotype of the *Formica* supergene
Sp: Polygyne-associated haplotype of the *Formica* supergene
