# Supplementary material for: A novel distribution of supergene genotypes is present in the socially polymorphic ant Formica neoclara
Source: BMC Ecol Evol. 2022 Apr 13;22:47. doi: 10.1186/s12862-022-02001-0 (PMC9006578; doi:10.1186/s12862-022-02001-0)
Supplement: Supplementary file 1 — Additional file 1: FigureS1. A Results of GEMMA analysis utilizing workers from colonies fromall regions, visualized via Manhattan plot. We used a linear mixed model withcolony social form as the independent variable. Each point represents anindividual SNP, with the corresponding chromosome on the x-axis and thenegative logarithm of the SNP p-value on the y-axis. Only one SNP, from chromosome 3, exceeds the significance level (Bonferroni corrected significance threshold: 1.52E−04;p-value: 7.26E−06). B Results of GEMMA analysis onworkers from colonies in Alberta only, to reduce the effect of underlyingpopulation structure on the GWAS, visualized via Manhattan plot. We used alinear mixed model with colony social form as the independent variable. Eachpoint represents an individual SNP, with the corresponding chromosome on thex-axis and the negative logarithm of the SNP p-value on the y-axis. Five SNPs,all from chromosome 3, are above the significance threshold (Bonferroni corrected significance threshold: 1.89E−04; p-values: 7.79E−07, three at 4.43E−07, and 3.79E−07). Figure S2. Stacked bar plot displaying genotypes of samples from colonies labeled as ambiguous in social form (three from Alberta, one from southern British Columbia). Each bar represents all samples from an individual colony. Genotype in relation to each individual worker, is indicated by color: green = Sm/Sm andbrown = Sm/Sp. Figure S3. Line graph showing the effects of missingness on the Huang estimator. Individual (red) and whole sample (black) relatedness values are represented as lines. Figure S4. Density plot showing pairwise relatedness (determined by PolyRelatedness) among all sequencedworkers of colonies from the Evan Thomas trailhead in Alberta, Canada. On average, workers from monogyne colonies are the most related, whereas intermediate colonies (“ambiguous” in Fig. 1) have a bimodal distribution offull siblings and individuals with intermediate relatedness values, andpolygyne colonies have th [file 12862_2022_2001_MOESM1_ESM.docx]

**A novel distribution of supergene genotypes is present in the socially polymorphic ant *Formica neoclara***

**Additional file 1**


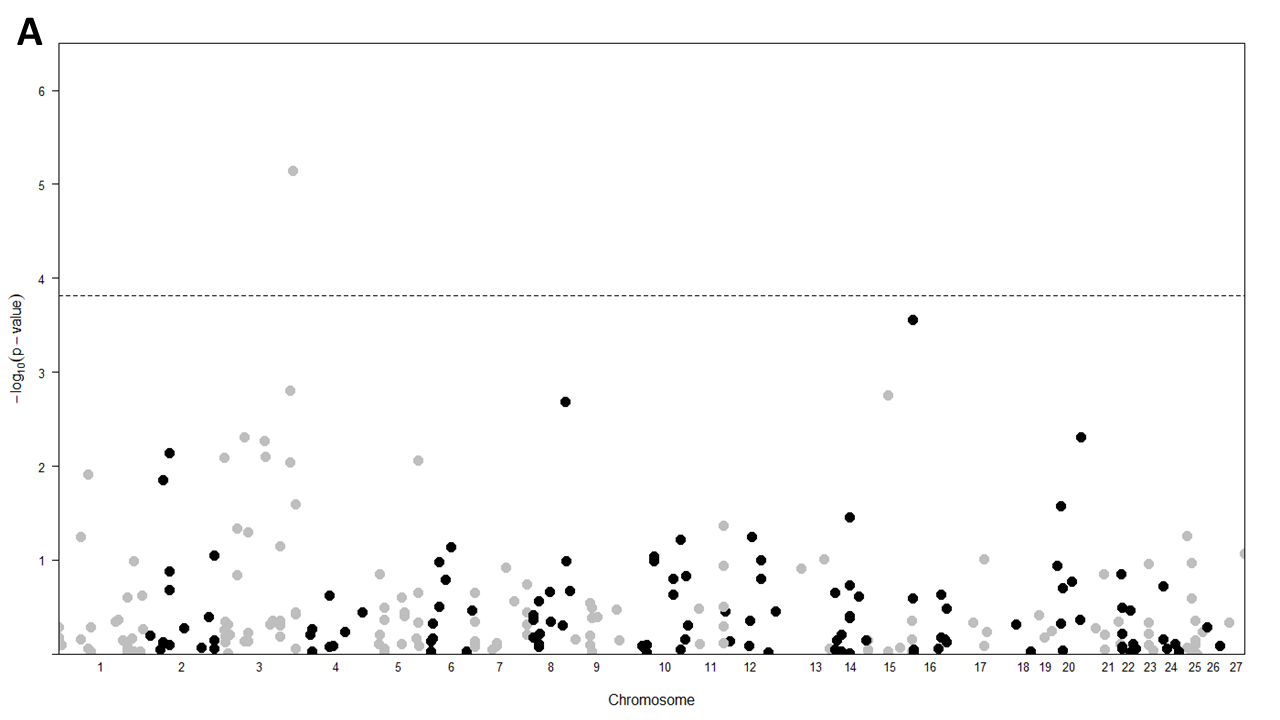


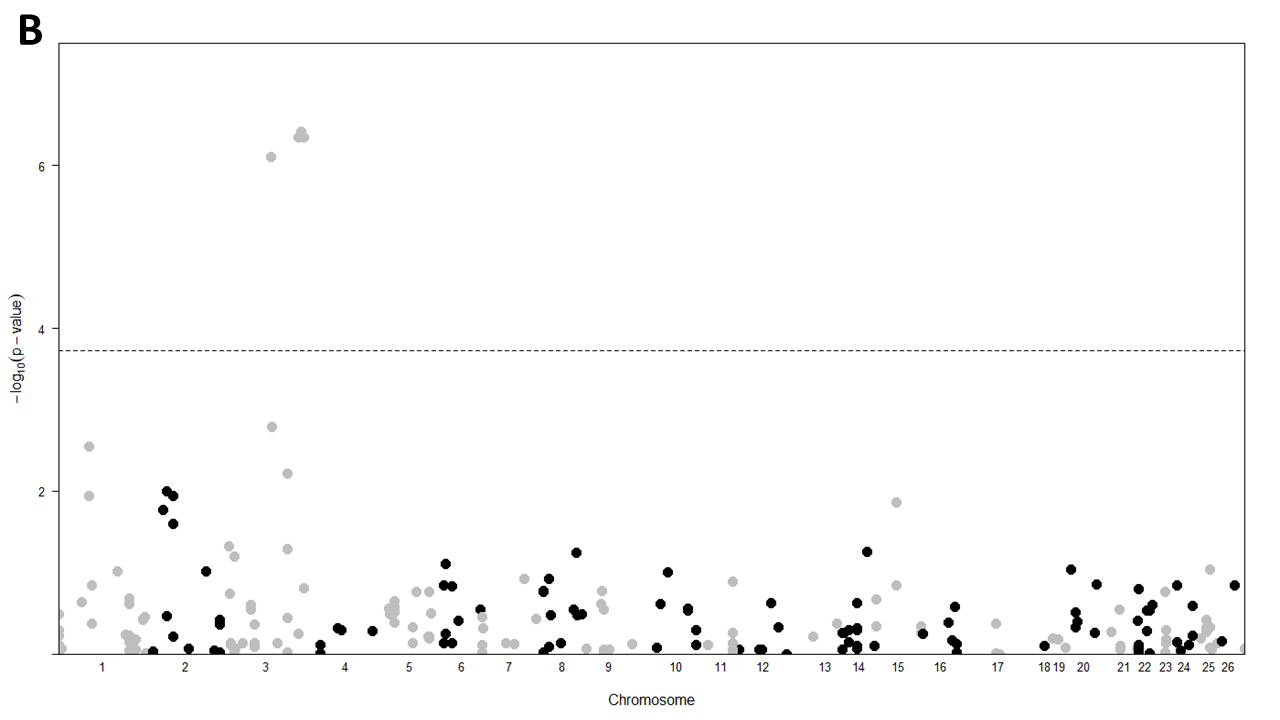


**Figure S1**. Panel **A**. Results of GEMMA analysis utilizing workers from colonies from all regions, visualized via Manhattan plot. We used a linear mixed model with colony social form as the independent variable. Each point represents an individual SNP, with the corresponding chromosome on the x-axis and the negative logarithm of the SNP p-value on the y-axis. Only one SNP, from chromosome 3, exceeds the significance level (Bonferroni corrected significance threshold: 1.52E-04; p-value: 7.26E-06). Panel **B**. Results of GEMMA analysis on workers from colonies in Alberta only, to reduce the effect of underlying population structure on the GWAS, visualized via Manhattan plot. We used a linear mixed model with colony social form as the independent variable. Each point represents an individual SNP, with the corresponding chromosome on the x-axis and the negative logarithm of the SNP p-value on the y-axis. Five SNPs, all from chromosome 3, are above the significance threshold (Bonferroni corrected significance threshold: 1.89E-04; p-values: 7.79E-07, three at 4.43E-07, and 3.79E-07).


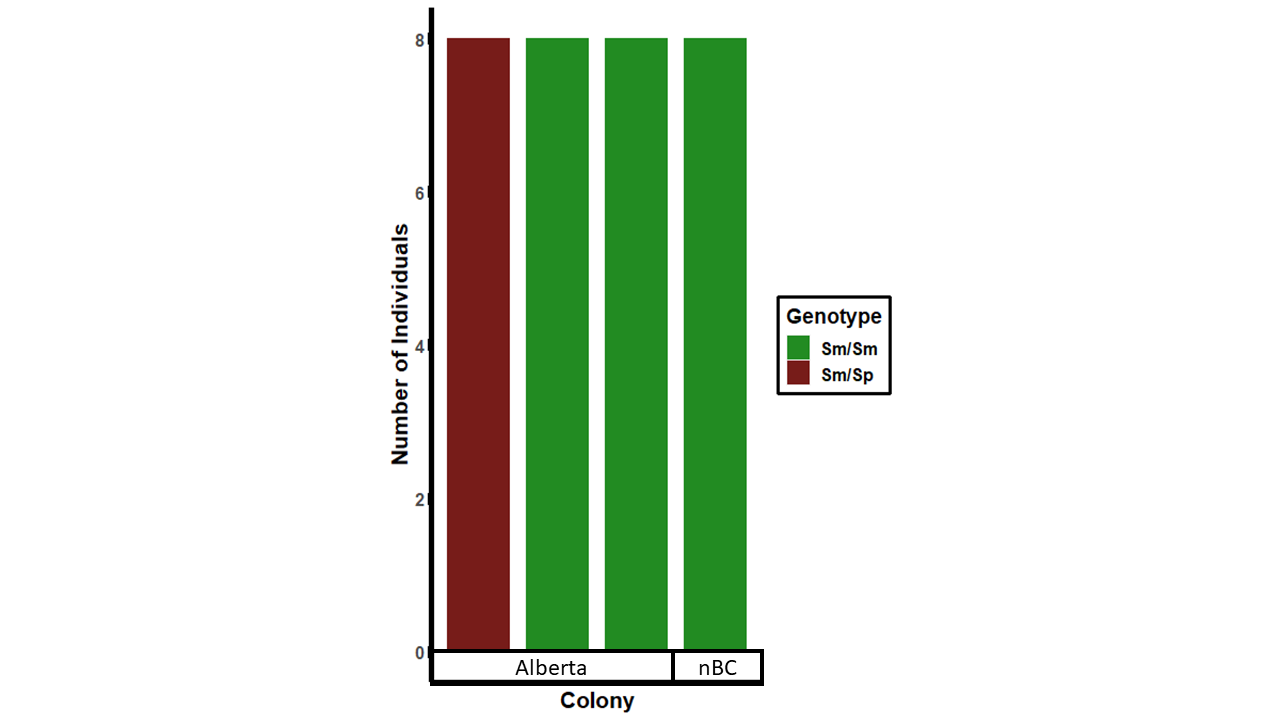


sBC

**Figure S2**. Stacked bar plot displaying genotypes of samples from colonies labeled as ambiguous in social form (three from Alberta, one from southern British Columbia). Each bar represents all samples from an individual colony. Genotype, in relation to each individual worker, is indicated by color: green = Sm/Sm and brown = Sm/Sp.


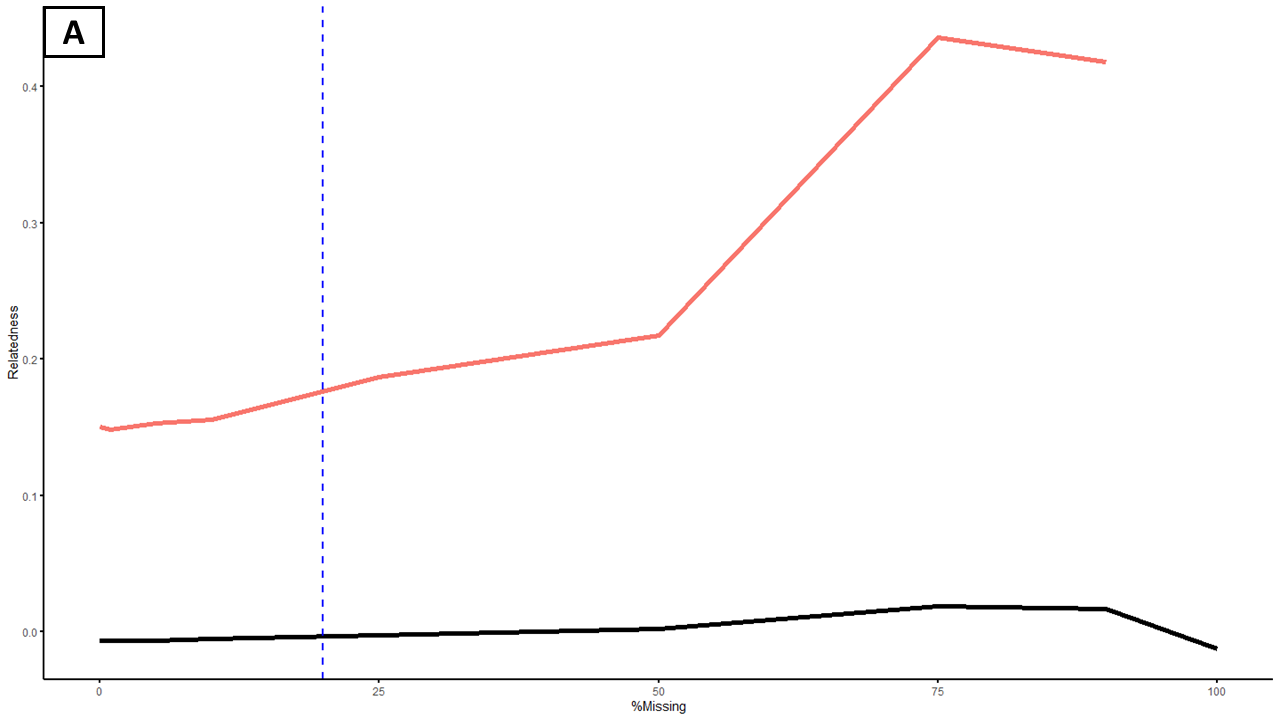


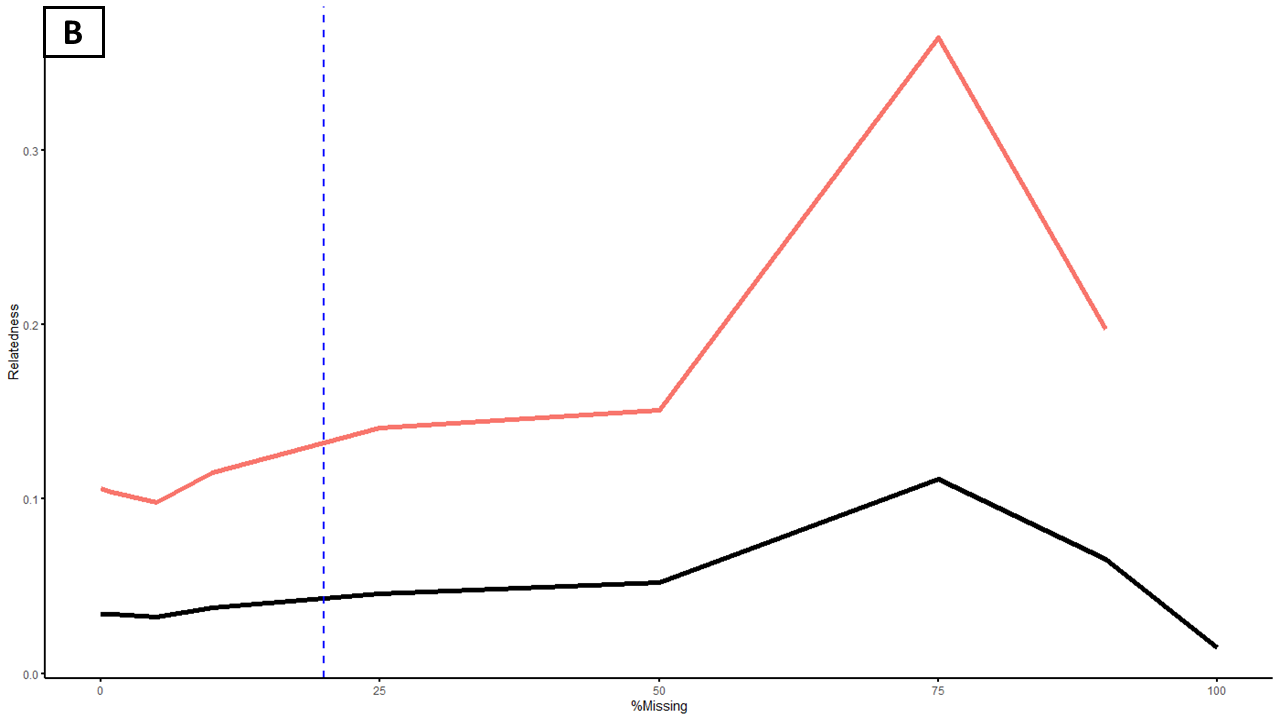


**Figure S3**. Line graph showing the effects of missingness on the Huang estimator. Individual (red) and whole sample (black) relatedness values are represented as lines. **Panel A** Samples used are 30 individuals with less than 1% missingness. **Panel B** Samples used are 8 individuals with less than 1% missingness. Missingness in one individual chosen at random (FRLC_6W8) was artificially inflated for varying increments. Notably, both individual and population pairwise relatedness values increase up to 75% missingness. Our missingness threshold of 20% is shown on each panel as the blue dashed line.

**
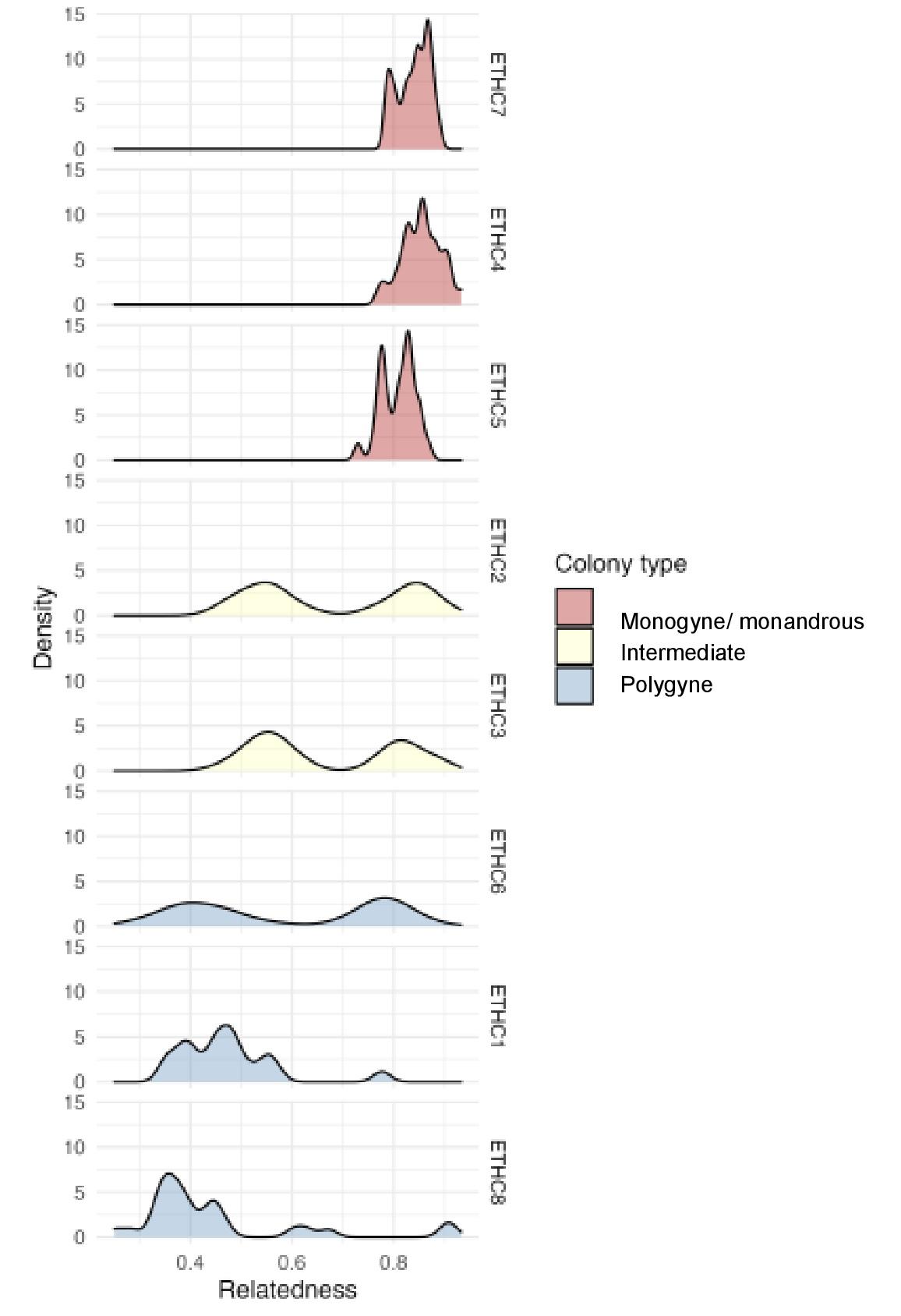
**

**Figure S4**. Density plot showing pairwise relatedness (determined by PolyRelatedness) among all sequenced workers of colonies from the Evan Thomas trailhead in Alberta, Canada. On average, workers from monogyne colonies are the most related, whereas intermediate colonies (“ambiguous” in Fig. 1) have a bimodal distribution of full siblings and individuals with intermediate relatedness values, and polygyne colonies have the lowest overall pairwise relatedness.


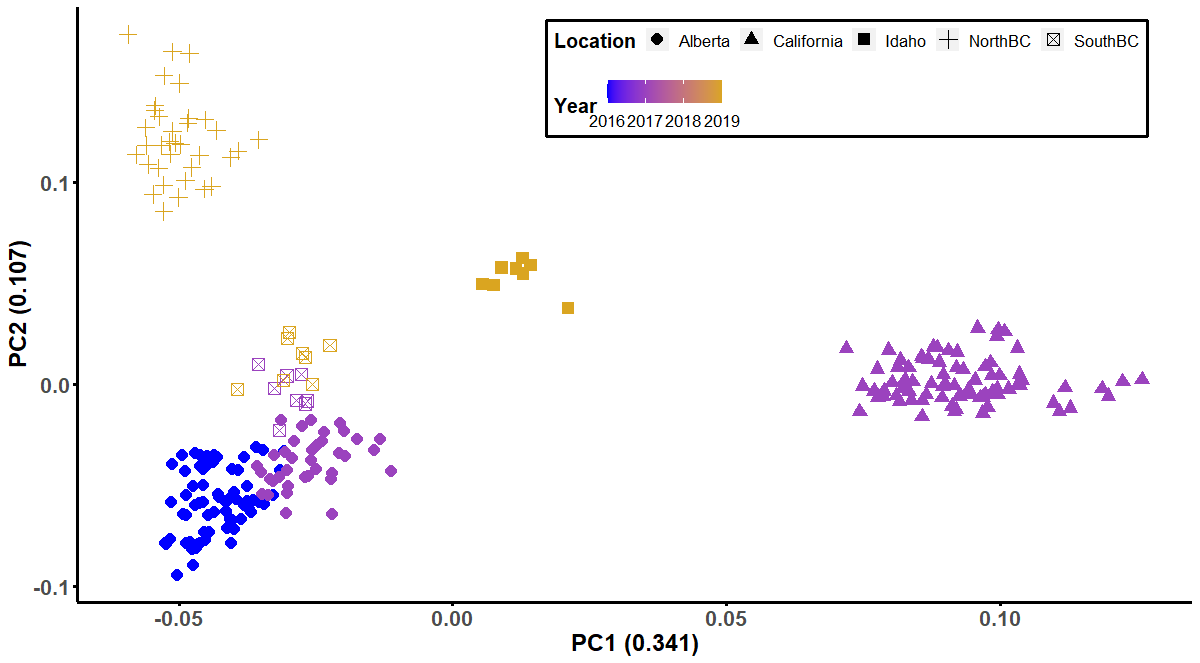


**Figure S5.** A principal component analysis utilizing all markers except those on chromosome 3 in individuals from colony samples, with the year of the respective batches color coded.

**Table S1**. Overview of consensus social form variables

| Colony | Region | Queen Number (inferred from *COLONY*) | Paternity Number *(inferred from COLONY)* | Opposing Homozygosity | Average Relatedness (Ajk estimator) | Colony KING | Colony  Huang | Colony IBD | SF |
| --- | --- | --- | --- | --- | --- | --- | --- | --- | --- |
| ETHC1 | Alberta | 2 | 6 | 43 | 0.361 | 0.211 | 0.468 | 0.468 | P |
| ETHC2 | Alberta | 1 | 2 | 18 | 0.526 | 0.301 | 0.679 | 0.678 | A |
| ETHC3 | Alberta | 1 | 2 | 20 | 0.516 | 0.312 | 0.668 | 0.668 | A |
| ETHC4 | Alberta | 1 | 1 | 0 | 0.689 | 0.424 | 0.854 | 0.854 | M |
| ETHC5 | Alberta | 1 | 1 | 0 | 0.667 | 0.384 | 0.808 | 0.807 | M |
| ETHC6 | Alberta | 1 | 2 | 33 | 0.451 | 0.290 | 0.587 | 0.583 | P |
| ETHC7 | Alberta | 1 | 1 | 4 | 0.848 | 0.401 | 0.839 | 0.839 | M |
| ETHC8 | Alberta | 3 | 5 | 46 | 0.372 | 0.193 | 0.443 | 0.447 | P |
| CALC1 | Alberta | 1 | 1 | 5 | 0.768 | 0.367 | 0.739 | 0.735 | M |
| CALC2 | Alberta | 1 | 6 | 41 | 0.416 | 0.219 | 0.429 | 0.431 | P |
| RVCC1 | Alberta | 1 | 2 | 13 | 0.553 | 0.333 | 0.601 | 0.593 | A |
| RVPC1 | Alberta | 1 | 1 | 3 | 0.673 | 0.346 | 0.769 | 0.770 | M |
| SYLC1 | Alberta | 1 | 1 | 1 | 0.902 | 0.369 | 0.744 | 0.745 | M |
| SYLC5 | Alberta | 1 | 1 | 1 | 0.739 | 0.382 | 0.766 | 0.764 | M |
| FRLC3 | NorthBC | 1 | 1 | 1 | 0.816 | 0.388 | 0.798 | 0.794 | M |
| FRLC4 | NorthBC | 1 | 2 | 20 | 0.583 | 0.343 | 0.679 | 0.676 | A |
| TOADC5 | SouthBC | 4 | 5 | 55 | 0.539 | 0.199 | 0.448 | 0.451 | P |
| TOADC7 | SouthBC | 4 | 6 | 51 | 0.525 | 0.179 | 0.434 | 0.414 | P |
| TOADC8 | SouthBC | 3 | 6 | 36 | 0.590 | 0.212 | 0.487 | 0.488 | P |
| TOADC9 | SouthBC | 2 | 5 | 46 | 0.557 | 0.222 | 0.505 | 0.436 | P |
| TOADC10 | SouthBC | 5 | 7 | 42 | 0.506 | 0.207 | 0.461 | 0.459 | P |
| SHEC17 | California | 1 | 1 | 2 | 1.08 | 0.337 | 0.784 | 0.781 | M |
| HROC2 | California | 2 | 4 | 30 | 1.06 | 0.224 | 0.662 | 0.666 | P |
| RGOC22 | California | 2 | 8 | 38 | 0.808 | 0.284 | 0.612 | 0.594 | P |
| RGOC25 | California | 3 | 3 | 18 | 0.677 | 0.299 | 0.609 | 0.598 | P |
| RGOC30 | California | 4 | 4 | 39 | 0.732 | 0.264 | 0.562 | 0.549 | P |
| RGOC31 | California | 4 | 7 | 25 | 0.751 | 0.275 | 0.579 | 0.563 | P |
| RGOC32 | California | 7 | 8 | 38 | 0.693 | 0.184 | 0.509 | 0.513 | P |
| RGOC33 | California | 5 | 7 | 45 | 0.714 | 0.245 | 0.535 | 0.525 | P |
| RGOC34 | California | 3 | 6 | 37 | 0.966 | 0.249 | 0.574 | 0.567 | P |
| RGOC36 | California | 2 | 7 | 38 | 0.759 | 0.165 | 0.467 | 0.458 | P |
| POC4 | Idaho | 2 | 2 | 6 | 0.887 | 0.358 | 0.832 | 0.823 | M |

Consensus social form variables include queen number (inferred by *COLONY*), opposing homozygosity, average colony relatedness estimators (Ajk, KING, Huang), and average colony identity by descent. If the majority of these values agree on one social form (SF, monogyne: M, polygyne: P, ambiguous: A, the colony is labeled as such.

**Table S2**. Sampling regions and their associated coordinate values

| Region | Locality | Locality Code | Latitude | Longitude | Colonies |
| --- | --- | --- | --- | --- | --- |
| Alberta | Evan Thomas Trailhead | ETC | 50.89 | -115.13 | 8 |
| Alberta | Rocky View County | RCV | 51.37 | -114.01 | 1 |
| Alberta | Kananaskis County RV Park | RVP | 50.89 | -115.15 | 1 |
| Alberta | Calgary | CAL | 51.05 | -114.08 | 2 |
| Alberta | Sylvan Lake | SYL | 52.31 | -114.09 | 2 |
| Northern British Columbia | Toad River | TOAD | 58.36 | -125.43 | 5 |
| Southern British Columbia | Fraser Lake | FRL | 54.06 | -124.84 | 3 |
| California | Royal Gorge | RGO | 39.32 | -120.37 | 8 |
| California | Hampshire Rock Campground | HRO | 39.31 | -120.5 | 1 |
| California | Sage Hen Field Station | SHE | 39.43 | -120.24 | 1 |
| Idaho | Pocatello | POC | 42.87 | -112.29 | 1 |

**Table S3.** PC axis weightings of variants on chromosome 3 (see also Figure 2). Weightings greater than 1 or less than -1 are highlighted in grey for each PC axis. The outliers from GWAS analyses are highlighted in green (Alberta only) and orange (GWAS of the full dataset).

|  |  | PC Axis Weighs | | |
| --- | --- | --- | --- | --- |
| Chromosome | Position (bp) | PC1 | PC2 | PC3 |
| Scaffold03 | 2144868 | 1.0004 | -0.32826 | 0.276591 |
| Scaffold03 | 2144874 | 1.22838 | -0.151904 | -0.24658 |
| Scaffold03 | 2144933 | 1.00105 | -0.337547 | 0.278446 |
| Scaffold03 | 2395760 | 0.817024 | 0.0708892 | 0.0528047 |
| Scaffold03 | 3458925 | -0.0442767 | -0.152665 | 1.40306 |
| Scaffold03 | 3482123 | 0.75331 | 1.40878 | 0.682458 |
| Scaffold03 | 4661076 | 0.315637 | 1.24557 | 1.14996 |
| Scaffold03 | 4661092 | 0.449315 | 1.64244 | 1.54006 |
| Scaffold03 | 5207945 | -0.728026 | 0.739209 | -1.70085 |
| Scaffold03 | 5207978 | -0.411925 | 0.725807 | -1.36368 |
| Scaffold03 | 5208011 | -0.964536 | 1.08616 | -0.901944 |
| Scaffold03 | 7705868 | 1.43151 | -0.175352 | -0.493708 |
| Scaffold03 | 7847618 | 1.4796 | 0.169451 | -0.312746 |
| Scaffold03 | 8545482 | -0.378403 | 0.369149 | -1.54717 |
| Scaffold03 | 9005614 | 0.582073 | -1.64586 | 0.0661412 |
| Scaffold03 | 10078334 | -0.871358 | 0.374321 | -1.05717 |
| Scaffold03 | 10078367 | 1.04034 | 1.48188 | 0.00421084 |
| Scaffold03 | 10078369 | 1.02593 | 1.58092 | -0.0036326 |
| Scaffold03 | 10148561 | 0.112842 | 1.47078 | -0.595122 |
| Scaffold03 | 11689706 | 1.63289 | -0.766107 | -0.445192 |
| Scaffold03 | 11689715 | -0.408464 | 0.265088 | 2.21584 |
| Scaffold03 | 12110992 | 1.08993 | 1.48176 | -0.179266 |
| Scaffold03 | 12477296 | 0.421355 | -1.32423 | -0.0021262 |
| Scaffold03 | 12541302 | 1.49868 | -0.238893 | -0.422726 |
| Scaffold03 | 12541325 | -0.487041 | 0.185668 | -0.229643 |
| Scaffold03 | 12541436 | 1.39437 | -0.19688 | -0.48575 |
